# Electrocardiogram derived QRS duration associations with elevated central aortic systolic pressure (CASP) in a rural Australian population

**DOI:** 10.1186/s40885-016-0039-8

**Published:** 2016-02-16

**Authors:** Yvonne Yin Leng Lee, Paul Reidler, Herbert Jelinek, Yung Seng Lee, Yuling Zhou, Brett D. Hambly, Joel McCabe, Slade Matthews, Honghong Ke, Hassan Assareh, Craig S. McLachlan

**Affiliations:** 1 Rural Clinical School, Faculty of Medicine, University of New South Wales, Room 327, Samuels Building, Sydney, NSW 2052 Australia; 2 School of Health Sciences, Charles Stuart University, Albury, Australia; 3 Department of Paediatrics, Yong Loo Lin School of Medicine, National University of Singapore, Singapore, Singapore; 4 Singapore Institute for Clinical Sciences, A*STAR, Singapore, Singapore; 5 Discipline of Pathology, Sydney Medical School, University of Sydney, Sydney, Australia; 6 Discipline of Pharmacology, Sydney Medical School, University of Sydney, Sydney, Australia; 7 Department of Cardiology, The First Affiliated Hospital of Guangxi Medical University, Nanning, China

**Keywords:** Aorta, Hypertension, Electrocardiography, Rural population

## Abstract

**Background:**

Prolonged electrocardiogram QRS durations are often present in hypertensive patients. Small increases in QRS duration serve as independent risk factors for both increased cardiovascular and all-cause mortality. Aortic stiffness is associated with increases in central aortic systolic blood pressure (CASP). However CASP and ECG QRS duration interactions have not been established in rural community populations. Our aims are to determine if QRS duration > 100 msec is associated with an elevated CASP measure in an Australian rural population.

**Methods:**

A retrospective cross sectional population was obtained from the CSU Diabetes Screening Research Initiative data base where 68 participants had both central aortic pressure recorded and ECG derived QRS duration. Central aortic pressure was determined by directly recording radial arterial tonometry and brachial cuff pressure (HealthStats, Singapore). Resting 12-lead electrocardiograms were obtained from each subject using a Welch Allyn PC-Based ECG system.

**Results:**

The population had a mean CASP of 137.8 mmHg, higher than previously reported in other population studies. In 8/68 subjects with a prolonged cardiac QRS duration >120 msec, CASP ranged from 129 mmHg – 182 mmHg. When subgroup analysis was stratified on the basis QRS duration <100 msec and ≥100 msec significant differences (*p* = 0.036) were observed for mean CASP, 130.6 mmHg ± 15.6 (SD) versus 140.6 mmHg ± 16.8 (SD), respectively.

**Conclusions:**

Our results suggest that an arbitrary CASP reading greater than a value 140 mmHg raises suspicion of a prolonged QRS duration. QRS durations ≥100 msec in an aging rural population are associated with higher CASP measures. Our results also suggest in aging Australian rural populations CASP is likely to be elevated, possibly due to age related aortic stiffening.

## Background

Electrocardiogram (ECG) derived QRS durations >100 msec and >120 msec have been both associated with hypertension induced increases in left ventricular (LV) mass [1–4]. Myocardial hypertrophy can alter LV tissue repolarisation and slowing of transmural conduction velocity [5, 6]. Slowed conduction of electrical impulses through the cardiac ventricles is marked by increases in QRS duration [6]. Cardiac hypertrophy may be associated with impaired coronary vasodilator reserve, decreased capillary density, reduced connexin-43 expression, and increased individual cardiac myocyte cell diameter, all of which can result in slowed conduction velocity [7, 8].

The pathology of LVH (left ventricular hypertrophy) can be associated with increased central systolic pressure and is related to aortic stiffness [9, 10]. Aortic stiffness has been shown across various models and human populations to increase cardiac afterload and reduce coronary vasodilator reserve [11]. Persistent increases in cardiac afterload can result in cardiac hypertrophic signalling and reduced coronary perfusion. Increased cardiac mass and cardiac cellular structural and electrical remodelling as a result of increased aortic stiffening would theoretically predict an increase in QRS duration associated with developed cardiac hypertrophy [1, 12].

Aortic elastic properties are relevant to increases in central aortic systolic pressures, particularly aortic stiffening. The aorta has an elastic buffering component often referred to as a Windkessel [10]. That is the aorta can store about 50 % of the left ventricular stroke volume during systole. In diastole, the stored volume is released from the elastic forces of the aortic wall to assist peripheral circulation [10]. The elastic resistance (or stiffness), increases with aging [12, 13], with an increase in systolic central and peripheral blood pressure [10]. Chronic stiffness of large conduit vessels can lead to cardiac hypertrophy [2, 14].

On the basis of ventricular-aortic coupling, central aortic systolic pressure (CASP) has emerged as a more accurate predictor of cardiovascular structural hypertrophy than brachial pressures and pulse pressures [15]. Pressure in the brachial artery differs from pressure in the ascending aorta due to amplification as the pressure pulse transits from central to peripheral arteries [15]. Clinically, peripheral pressures do not always reflect central aortic systolic stiffening [15, 16]. Importantly, the degree of amplification is not fixed. CASP varies between individuals depending upon age, cardiovascular disease and/or drug therapy [15–17]. Central aortic pressure may be derived non-invasively by accurate registration, capture and calibration of a peripheral artery pressure waveform using an externally applied arterial sensor with or without a brachial pressure cuff reading [16]. The captured pressure waveform undergoes mathematical transformations to derive central aortic pressure, the validity of these processes having been established during direct invasive catheter measurements of the ascending aorta [17].

Considering the pathological implications for increased aortic stiffness and impaired ventricular aortic coupling, it would be logical to suggest that increased central aortic systolic pressure would be associated with increased QRS duration. However, to date there have been no studies in the literature exploring this association in a patient series using a CASP device. Our present study explores differences in ECG derived QRS duration in 68 rural subjects with and without type 2 diabetes and with and without hypertension. Interactions with age and CASP are modelled as factors that could influence prolonged QRS duration.

## Methods

Human ethics clearance was obtained from Charles Stuart University (CSU) and written informed consent from the patients involved in the present study. A retrospective cross sectional population was obtained from the CSU Diabetes Screening Research Initiative (DiScRi) data base where 68 participants had both central aortic pressure recorded and ECG derived QRS duration. Subjects were excluded if pregnant, non-ambulatory, cognitively impaired, or had a history of heart failure. Two blood pressure (BP) readings at the upper arm were taken with the patient in a supine position, after resting for 30 min, and the average recorded. Central aortic pressure was determined using A-PULSE CASPro®, by directly recording radial arterial tonometry and brachial cuff pressure (HealthStats, Singapore) [15]. The A-PULSE CASPro® algorithm for determining CASP has been previous validated, for example a comparison made of CASP estimated with the B-Pro device and SphygmoCor (as the “gold standard” reference device) in 104 healthy Caucasians demonstrated an correlation coefficient (*r* = 0.937) [18]. There is also excellent agreement (r^2^ = 0.98, *p* < 0.001) between directly measured aortic root systolic pressures using a Millar’s catheter at cardiac catheterization versus NPMA-CASP HeathSTATS algorithm [15].

Resting 12-lead electrocardiograms were obtained from each subject using a Welch Allyn PC-Based ECG system as previously described [4]. This permitted automated QRS duration calculation from a 10 s ECG trace. Statistics were presented as means and standard deviation (SD); Pearson product–moment correlation analysis and regression analysis was used to explore age interactions with CASP and QRS duration. Statistical significance was defined as *p* < 0.05.

## Results

The demographics for the 68 subjects are shown in Table 1. The mean age was 69.4 years and 8 subjects out of 68 subjects had QRS durations greater than 120 msec. Mean CASP levels for the study population were 138 mmHg ± 17 mmHg. The data was divided on patients self-reporting being hypertensive or not. Those diagnosed with hypertension were being treated. The consequences of being treated or not being treated with hypertension medications revealed no significant differences for mean QRS duration (104 ± 17 msec vs 107 ± 15 msec (BP treated) or mean CASP levels (134 ± 20 mmHg vs 139 ± 14 mmHg (BP treated).

**Table 1:** Population demographics and summary statistics

	Total cohort (*N* = 68)	
	N or mean (SD)	%
Age, years	69.4 (9.7)	
Gender		
Female	42	61.8
Male	26	38.2
Hypertension	39	60
Cardiovascular disease	12	18.8
Diabetes	22	32.4
BMI	27.9 (4.6)	
QRS (ms)	106.3 (15.7)	
< 120	59	88.1
> 120	8	11.9
Peripheral SBP (mmHg)	133.5 (16.4)	
< 110	4	5.9
> 110	64	94.1
< 120	13	19.1
> 120	55	80.9
Peripheral DBP (mmHg)	77 (8.4)	
CASP (mmHg)	137.8 (17.2)	
< 110	4	5.9
> 110	64	94.1
Central SBP (mmHg)	146.1 (17.5)	
Central DBP (mmHg)	85.9 (10.2)	
Central PP (mmHg)	101.3 (10.7)	

*CASP* central aortic systolic pressure, *SD* standard deviation

**Fig. 1:**
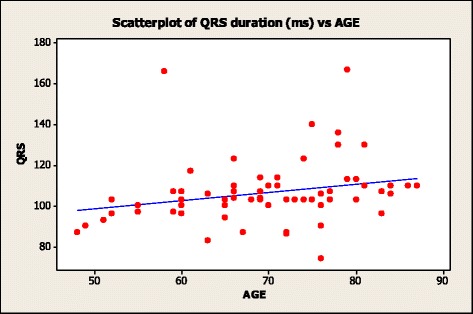
Scatter plot of subjects Age and QRS duration. The regression line is shown in blue; regression analysis R-Sq = 6.2 %; *p* = 0.043

**Fig. 2:**
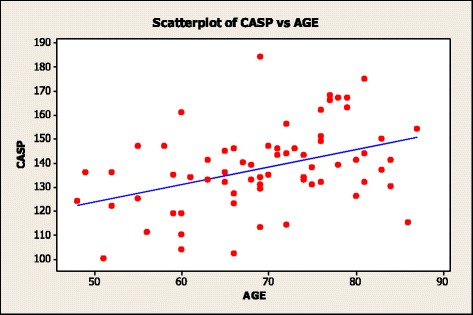
Scatter plot of subjects CASP and AGE. Regression line is shown in blue; regression analysis R-Sq = 13.4 %; *p* = 0.003

Pearson’s correlation analysis showed a modest but significant association between QRS duration and CASP (*r* = 0.25; *p* = 0.039). Both QRS duration (*r* = 0.25; *p* = 0.043) (Fig. 1) and CASP (*r* = 0.41; *p* = 0.001) (Fig. 2) had a significant but modest association with age. When we stratified subpopulations on the basis of QRS duration <100 msec and ≥100 msec significant differences (*p* = 0.036) were observed for mean CASP, 130.6 ± 15.6 (SD) versus 140.6 ± 16.8 (SD), respectively. The mean CASP in subjects with a QRS duration > 120 msec was 145.25 mmHg ± 14.9(SD). Of the subjects with a QRS duration >120 msec only 1 subject had a QRS duration less than 130 msec, and 4/8 subjects had a QRS duration >140 msec. Current guidelines have defined a suspicion for left bundle branch block (LBBB) using a QRS duration of ≥130 msec in women and a QRS duration of ≥ 140 msec in men [19]. Four subjects met the definition for suspicion of LBBB. The average CASP for these 4 cases was 148 ± 14 mmHg. When these 4 suspected LBBB cases were removed, the statistical difference in mean CASP stratified on the basis of QRS duration <100 msec (130.6 ± 15.6 mmHg) and ≥100 msec (140 ± 14 mmHg) was not significant at *p* = 0.051.

## Discussion and conclusions

In previous published population studies with age ranges 40 - 65 we have noted a median CASP of 115 mmHg [9]. We arbitrarily set a conservative CASP cut-off of 110 mm/Hg as being within normal limits for healthy aging individuals [10]. Therefore, we defined a CASP >110 mmHg as being abnormal. The mean CASP for our cohort was 137.8 mmHg – only 3/68 subjects in our cohort had a CASP less than 110 mm/Hg (Table 1). When we explored CASP readings in the 8 subjects with a QRS duration >120 msec we found that the mean CASP to be 145 mm/Hg (range 127–167 mmHg) (Table 2).

**Table 2:** Summary data for 8 subjects QRS > 120 msec

	Total cohort (*n* = 8)	
	N or mean (SD)	%
Age, years	73.6 (7.8)	
Gender		
Female	5	62.5
Male	3	37.5
Hypertensive	4	50
Cardiovascular disease	0	
Diabetes	1	12.5
BMI (median, range)	29.6 (4.9)	
Peripheral SBP (mmHg)	143.5 (11.2)	
> 110	8	100
Peripheral DBP (mmHg)	82.4 (5.3)	
CASP (mmHg)	145 (14.9)	
> 110	8	100
Central SBP (mmHg)	154.3 (18.4)	
Central DBP (mmHg)	89.6 (8.4)	
Central PP (mmHg)	106.6 (8.4)	

Four cases with QRS duration > 120 msec were associated with LBBB risk using the QRS duration cut-offs described in the Strauss criteria [19]. We did not have access to the original ECG traces, only the automated QRS durations for retrospective analysis, thus mid-QRS notching in the leads to confirm LBBB using the Strauss criteria were not available [19]. On the other hand we question whether LBBB was present, given that CASP was elevated for each case. For example, one case study of interment LBBB, when compared to normal conduction, LBBB was associated with a 20 mmHg decline in systolic pressures in the left ventricle, central aorta and radial artery [20].

CASP increases by approximately 3–4 mmHg per decade of life [15], while the difference between our cohort overall and those patients with a prolonged QRS was estimated to be double this value at 7 mmHg per decade of life. Our findings demonstrate that in an Australian aging rural population, mean CASP are greater (138 mmHg) than those reported in other published community studies with mixed healthy and chronic cardiovascular disease participants (115–130 mmHg) [21–23]. However, the mean CASP for our mixed population (138 mmHg) are not dissimilar to central aortic systolic pressures reported in patients undergoing cardiac catheter coronary interventions (139.6 ± 1.4 mmHg) [24]. Surprisingly, despite the mean CASP in our mixed, aging, rural population being higher than previous studies, elevated CASP in our aging cohort were rarely associated with an increase in QRS width greater than 120 msec. We observed no differences in QRS durations in those being treated for diagnosed hypertension and those not being treated (i.e. subjects without known hypertension).

Like most Australian rural populations, patients are medically undertreated for blood pressure or are non compliant with therapy. Previously, we have identified a low usage of ACE inhibitors in the rural area of Albury [25]. ACE inhibitors have been shown to reduce aortic stiffening, independent of their blood pressure lowering effects [26]. We suggest that blood pressure medication other than ACE inhibitors may have favourably influenced ventricular remodelling. For example, it has been shown that that carvedilol results in a favourable pulse pressure amplification and augmentation index by increasing arterial compliance and reducing the magnitude of wave reflection in hypertension [27]. Our cohort was older (median 70 years) than previous published data on median CASP levels and this may explain in part why our mean CASP levels were higher than in previous reports.

Additionally our aging cohort would have other inflammatory chronic conditions that would also increase vascular stiffness [28–30]. We suggest because of the likely higher burden of inflammatory conditions associated with more advanced aging, it is not surprising to find an elevated CASP in our cohort.

We acknowledge our sample size was small and therefore, the validity of the signal averaged ECG QRS duration in association with CASP needs to be verified in a larger study. Our study is cross-sectional in design and therefore we are careful not to infer causality. In summary, our results suggest that an arbitrary CASP reading greater than a value 140 mmHg raises suspicion of a prolonged QRS duration >120mesc. QRS durations ≥100 msec in an aging rural population are associated with higher CASP measures. Our results also suggest in aging Australian rural populations CASP is likely to be elevated, possibly due to age related aortic stiffening [30] and increased CASP does not always equate with a prolonged QRS duration.
